# Evaluation of liver function using gadolinium-ethoxybenzyl-diethylenetriamine pentaacetic acid enhanced magnetic resonance imaging based on a three-dimensional volumetric analysis system

**DOI:** 10.1007/s12072-018-9874-x

**Published:** 2018-06-02

**Authors:** Masashi Kudo, Naoto Gotohda, Motokazu Sugimoto, Tatsushi Kobayashi, Motohiro Kojima, Shinichiro Takahashi, Masaru Konishi, Ryuichi Hayashi

**Affiliations:** 1 Department of Hepatobiliary and Pancreatic Surgery, National Cancer Center Hospital East, 6-5-1 Kashiwa-no-ha, Kashiwa, Chiba 277-8577 Japan; 2 Department of Diagnostic Radiology, National Cancer Center Hospital East, Kashiwa, Japan; 3 Department of Pathology and Clinical Laboratory, National Cancer Center Hospital East, Kashiwa, Japan; 4 Course of Advanced Clinical Research of Cancer, Juntendo University Graduate School of Medicine, Tokyo, Japan

**Keywords:** Magnetic resonance imaging, Gadolinium-ethoxybenzyl-diethylenetriamine pentaacetic acid, Three-dimensional volumetric analysis system, Liver function, Liver steatosis

## Abstract

**Background:**

Magnetic resonance imaging with gadolinium-ethoxybenzyl-diethylenetriamine pentaacetic acid (EOB-MRI) is a diagnostic modality for liver tumors. Three-dimensional (3D) volumetric analysis systems using EOB-MRI data are used to simulate liver anatomy for surgery. This study was conducted to investigate clinical utility of a 3D volumetric analysis system on EOB-MRI to evaluate liver function.

**Methods:**

Between August 2014 and December 2015, 181 patients underwent laboratory and radiological exams as standardized preoperative evaluation for liver surgery. The liver-spleen contrast-enhanced ratio (LSR) was measured by a semi-automated 3D volumetric analysis system on EOB-MRI. First, the inter-evaluator variability of the calculated LSR was evaluated. Additionally, a subset of liver surgical specimens was evaluated histologically by using immunohistochemical staining. Finally, the correlations between the LSR and grading systems of liver function, laboratory data, or histological findings were analyzed.

**Results:**

The inter-evaluator correlation coefficient of the measured LSR was 0.986. The mean LSR was significantly correlated with the Child–Pugh score (*p* = 0.014) and the ALBI score (*p* < 0.001). Significant correlations were also observed between the LSR and indocyanine green retention rate at 15 min (*r* = − 0.601, *p* < 0.001), between the LSR and liver fibrosis stage (*r* = − 0.556, *p* < 0.001), and between the LSR and liver steatosis grade (*r* = − 0.396, *p* < 0.001).

**Conclusion:**

The LSR calculated by a 3D volumetric analysis system on EOB-MRI was highly reproducible and was shown to be correlated with liver function parameters and liver histology. These data suggest that this imaging modality can be a reliable tool to evaluate liver function.

**Electronic supplementary material:**

The online version of this article (10.1007/s12072-018-9874-x) contains supplementary material, which is available to authorized users.

## Introduction

After major hepatectomy, the reported incidence of liver failure is 3–8%, and the reported rate of mortality associated with liver failure is approximately 5% [1]. Preoperative precise assessment of liver function, extent of resection, and estimated remnant liver volume is important to minimize the risks associated with liver surgery.

Gadolinium ethoxybenzyl diethylenetriamine pentaacetic acid (Gd-EOB-DTPA) is a liver-specific magnetic resonance imaging (MRI) contrast agent. MRI using Gd-EOB-DTPA (EOB-MRI) is widely used to detect and characterize hepatocellular carcinoma or metastatic liver tumors [2]. Gd-EOB-DTPA has a well-known metabolic pathway, and several studies have suggested that EOB-MRI is useful to evaluate liver function [3, 4]. The liver-to-spleen signal intensity ratio (LSR) on EOB-MRI has been used as a parameter to assess liver function [5–8], but the conventional method to measure the LSR using two-dimensional (2D) regions of interest might be affected by sampling errors or inter-evaluator variability [9].

In a related field, three-dimensional (3D) volumetric analysis systems are being used to simulate liver anatomy for surgery. Using these systems, inter-evaluator variability is expected to be reduced, as signal intensity from the whole liver can be included in a semi-automatic analysis. There have been no previous clinical studies in which the 3D volumetric analysis system was used to measure the LSR on EOB-MRI. Therefore, the objective of this study was to evaluate the variability of the calculated LSR of EOB-MRI using a 3D volumetric analysis system, and to investigate the correlations between the LSR and liver function parameters or histological findings.

## Materials and methods

### Patients

Between August 2014 and December 2015, a total of 304 consecutive patients underwent laboratory and radiological examinations as preoperative evaluations in consideration of liver surgery at the National Cancer Center Hospital East, Japan. Of the 304 patients, 123 were excluded for the following reasons: contraindications to EOB-MRI (*n* = 29), inconsistent MRI acquisition technique (*n* = 52), incomplete laboratory data (*n* = 3), or prior splenectomy (*n* = 1). Thirty-eight patients who had undergone portal vein embolization were also excluded because the degree of liver enhancement from Gd-EOB-DTPA could depend considerably on the portal vein flow.

**Fig. 1:**
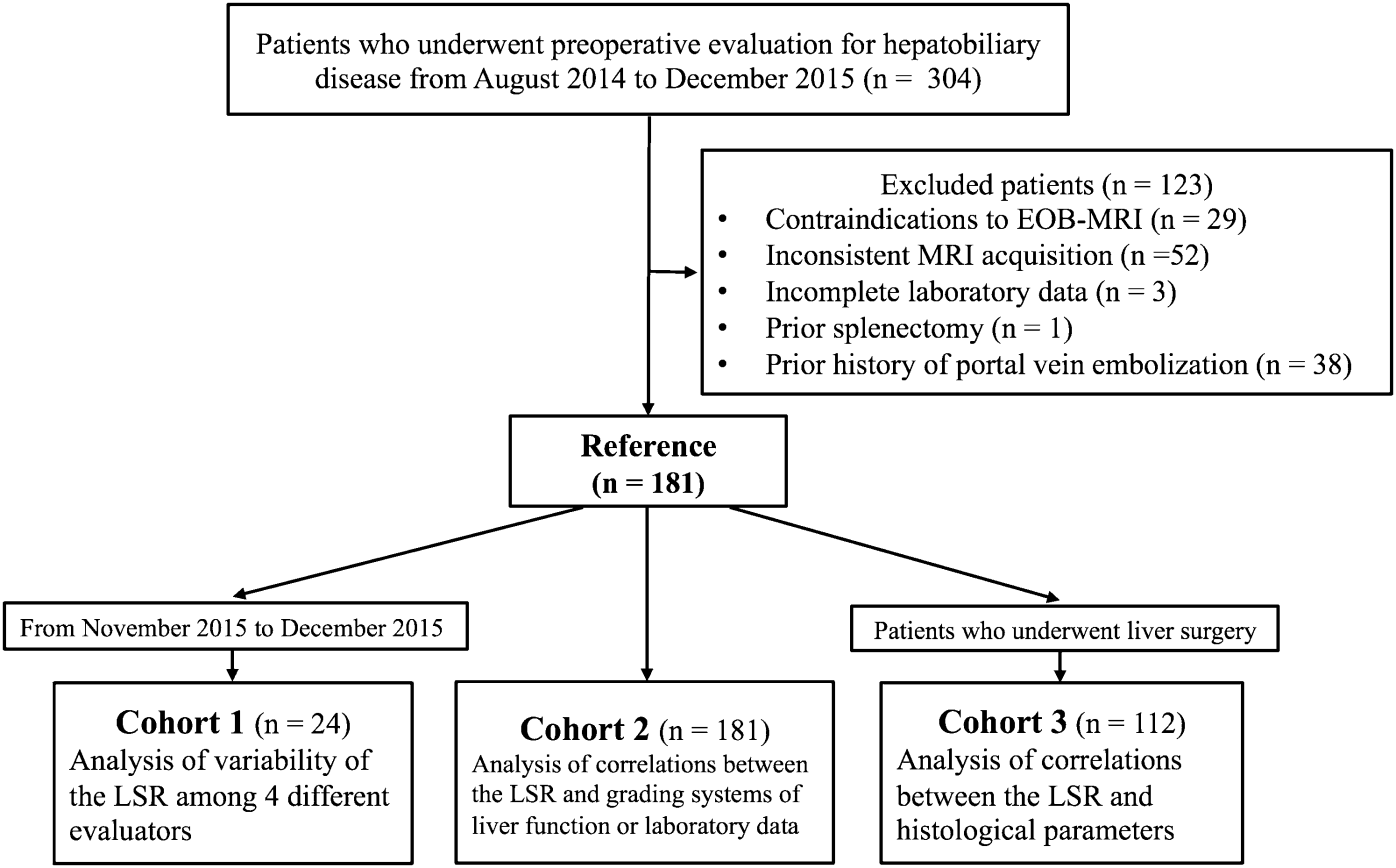
Flow-diagram describing the patient cohorts in this study. *Gd-EOB-DTPA* gadolinium ethoxybenzyl diethylenetriamine pentaacetic acid; *LSR* liver-to-spleen signal intensity ratio

The remaining 181 patients who underwent EOB-MRI of the liver using a standardized imaging technique were used for the analysis in this study. Among them, 24 consecutive patients who underwent preoperative evaluation from November 2015 to December 2015 were used to examine the variability of the LSR among four different evaluators (patient cohort 1). Then, all the 181 patients were used to analyze the correlations between the LSR and grading systems of liver function or laboratory data (patient cohort 2). Finally, a subgroup of all 112 patients who underwent liver resection was used to analyze the correlations between the LSR and histological parameters (patient cohort 3). The patient flow chart of this study is shown in Fig. 1.

### MRI protocol

All MRI studies were performed using one of the two 3.0 T scanners at our institution (Achieva or Ingenia, Philips Medical Systems, Amsterdam, Netherlands). Contrast-enhanced 3D fat-suppressed T1-weighted images were obtained 20 min after intravenous administration of Gd-EOB-DTPA for hepatobiliary phase imaging, using the following parameters: repetition time (TR) 4 ms, echo time (TE) 2 ms, flip angle 10°, slice thickness 4.6 mm, matrix size 512 × 512 (Achieva), and TR 3 ms, TE 2 ms, flip angle 10°, slice thickness 4.6 mm, matrix size 480 × 480 (Ingenia). Gd-EOB-DTPA was administered at a dose of about 0.1 mL/kg, by rapid intravenous bolus injection using a power injector (SONIC SHOT GX, NEMOTOKYORINDO, Tokyo, Japan), at a rate of 2 mL/s.

### MRI data analysis

**Fig. 2:**
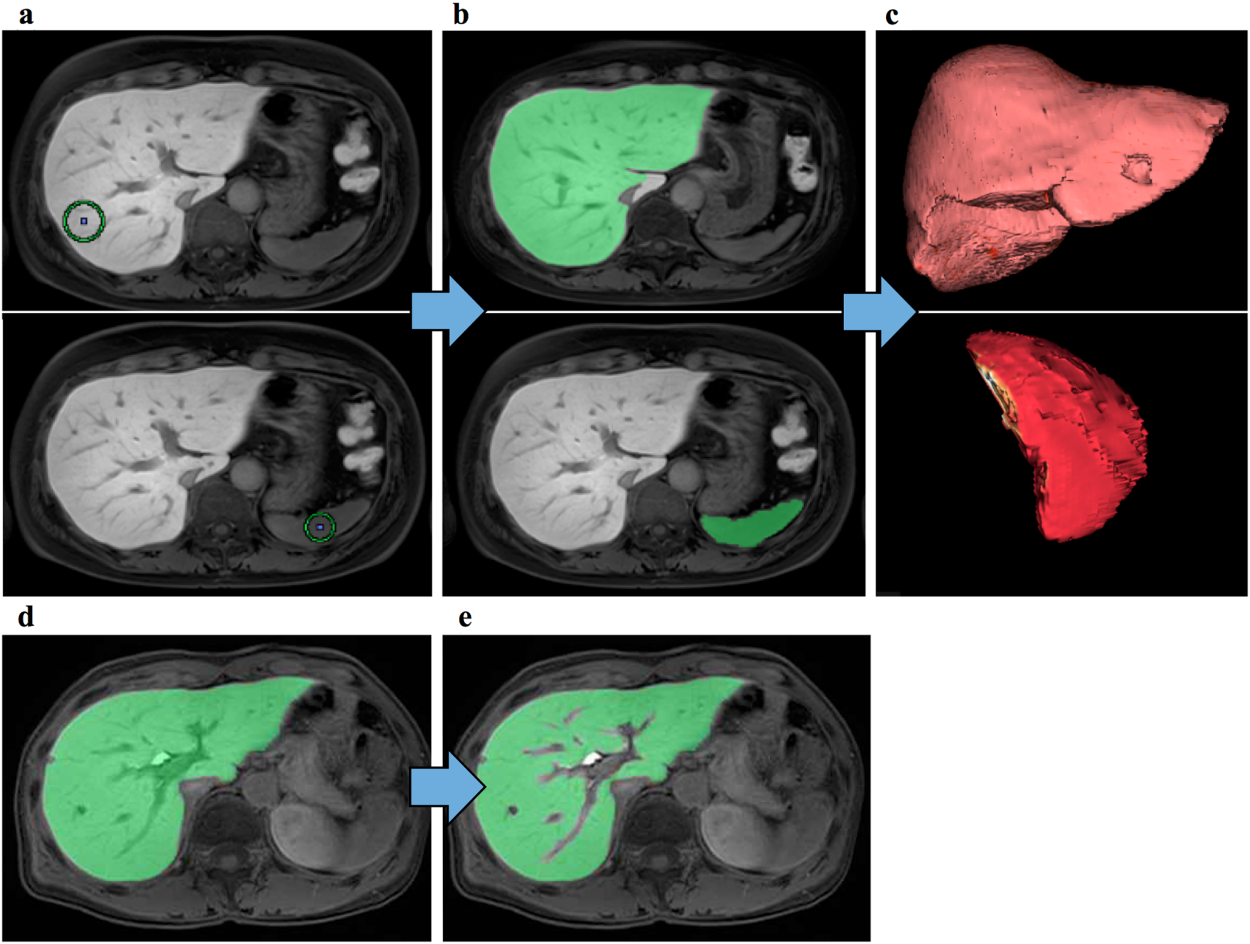
Image analysis using the three-dimensional volumetric system. **a** The investigator placed a small volume of interest (VOI) in the liver parenchyma. **b** The liver parenchyma was semi-automatically extracted from the initial VOI seed. **c** The three-dimensional liver volume was extracted, and the average liver signal intensity was calculated semi-automatically. **d** Liver parenchyma extracted before subtracting vascular structures. **e** Liver parenchyma after subtracting vascular structures, such as portal veins and hepatic veins, and intrahepatic bile ducts

The LSR was calculated using images from the 20 min-delayed hepatobiliary phase, with the 3D volumetric analysis system SYNAPSE VINCENT (Fujifilm Medical, Tokyo, Japan), by a single investigator (Ma.Ku.), under supervision of an experienced radiologist (T.K.). First, the investigator placed small operator-defined volumes of interest (VOIs), one in the liver and another in the spleen parenchyma, avoiding vessels and tumors (Fig. 2a). Second, the liver and spleen parenchyma were semi-automatically extracted using the image-processing algorithm (Fig. 2b). Finally, the LSR was calculated as the average liver parenchyma signal intensity (Fig. 2c) divided by the average spleen parenchyma signal intensity. Using the patient cohort 1, the variability of the calculated LSR among four evaluators (surgeons with 7–11 years of clinical experience) was analyzed. Then, since inclusion of extrahepatic parenchymal tissue, such as portal and hepatic veins, intrahepatic bile ducts, cysts, and tumors, might affect the average liver signal intensity, these structures were subtracted manually by the investigator (Fig. 2e). Additionally, the vascular subtraction LSR (vsLSR) was calculated, and its correlation with the LSR was analyzed using cohort 1 (Fig. 1).

### Grading systems of liver function and laboratory data

The following laboratory parameters were obtained preoperatively and within a month before or after EOB-MRI were collected from the patients’ medical records: indocyanine green retention rate at 15 min (ICGR15), white blood cell count, platelet count, prothrombin activity, and serum levels of hemoglobin, albumin, total bilirubin, aspartate aminotransferase, alanine aminotransferase, and creatinine. Grading systems of liver function, such as the Child–Pugh score and the albumin-bilirubin (ALBI) score, were analyzed for the correlation with the LSR. The Child–Pugh score was graded into A, B, or C, using five variables, including bilirubin, albumin, prothrombin, ascites status, and degree of encephalopathy. The ALBI score was calculated as follows:

$$ -0.085 \times (\text{albumin}\, \text{g/l}) + 0.66 \times \log (\text{total} \, \text{bilirubin} \, \upmu \text{mol/l}), $$

and categorized into the following three grades:

$$ \begin{aligned} < -2.60, \text{ALBI}\; 1; > 2.06 \; \text{to} -1.39, \text{ALBI} \; 2; > -1.39, \text{ALBI}\; 3. \end{aligned}$$

Using patient cohort 2, the correlations between the LSR and grading systems of liver function or other laboratory data were analyzed (Fig. 1).

### Histological analysis of the surgical specimen

**Fig. 3:**
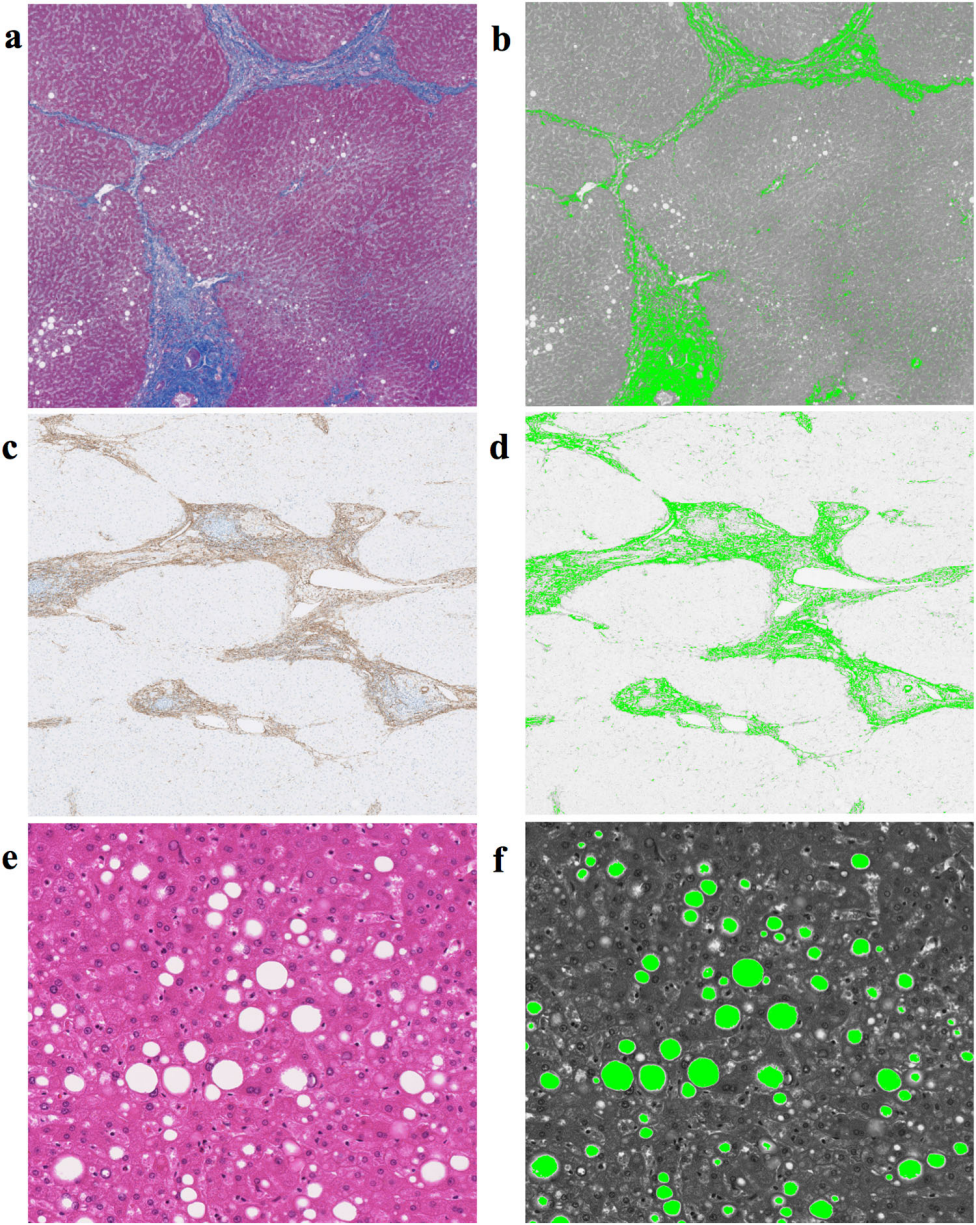
Areas of fibrosis and steatosis were calculated using morphometric analysis of color-detecting algorithm (WinROOF software, version 6.5; MITANI Corporation, Tokyo, Japan). **a** Azo carmine aniline blue (AZAN) and **c** smooth muscle actin (SMA) stain. The AZAN/SMA-positive area was determined using a color-detecting algorithm and is represented as bright green in **b** and **d**. The ratio of fibrosis (ROF) was calculated as the percentage area of the entire field and the AZAN/SMA-positive area. Hematoxylin–eosin stain of fatty liver (**e**). Fat droplets were determined using a color-detecting algorithm and are represented as bright green in image (**f**). The ratio of steatosis (ROS) was calculated as the percentage area of the entire field and the fat droplets area

From the 181 studied patients, 112 patients underwent liver resection (patient cohort 3) (Fig. 1). Their surgical specimens of non-tumoral liver tissues were fixed in formalin and embedded in paraffin. Then, 4-µm-thick sections were stained by hematoxylin and eosin (HE), azo carmine aniline blue (AZAN), and smooth muscle actin (SMA) (Fig. 3). The specimen slides were scanned using the NanoZoomer 2.0 system (Hamamatsu Photonics, Hamamatsu, Japan), and morphometric analysis was performed using the WinROOF 6.5 image processing software program (MITANI Corporation, Tokyo, Japan). Histological analyses were performed by a single investigator (Ma.Ku.), under supervision of an experienced pathologist (Mo.Ko.).

Liver fibrosis was assessed in three ways. First, using HE-stained slides, the fibrosis stage was morphologically categorized by the METAVIR scoring system as follows: F0, no fibrosis; F1, portal fibrosis without septa; F2, few septa; F3, numerous septa without cirrhosis; F4, cirrhosis [10]. Second, the ratio of fibrosis (ROF) on AZAN-stained slides (ROFazan) was quantified using morphometric analysis from a color-detecting algorithm (Fig. 3b), using the same technique as reported in a previous study [11]. Third, the ROF on SMA-stained slides (ROFsma) was quantified in a similar manner (Fig. 3d).

Liver steatosis was also assessed in two ways. First, the steatosis grade was morphologically categorized as in Kleiner et al. [12]: grade 0, < 5%; grade 1, 5–33%; grade 2, 34–66%; grade 3, > 67%. Second, the ratio of droplet steatosis (ROS) was quantified using morphometric analysis from the color-detecting algorithm (Fig. 3f).

### Statistical analysis

Statistical analyses were performed using JMP (version 12.0.10; SAS Institute, Cary, NC). Ebel’s intraclass correlation coefficients were used to evaluate the inter-evaluator variability of the LSR and the correlation between the LSR and vsLSR in patient cohort 1. Correlations between the LSR and the clinicopathological factors were assessed by the standard Pearson’s correlation coefficient or Spearman’s rank correlation coefficient in patient cohorts 2 and 3. The correlations between the LSR and fibrosis stage or steatosis grade were evaluated using pairwise comparisons with the Mann–Whitney test in patient cohort 3. Two-sided *p* values of less than 0.05 were considered indicative of significance.

## Results

### Patients’ demographics

The median age was 70 (range, 39–90), and 130 (72%) of 181 patients were male. The diagnoses were as follows: hepatocellular carcinoma in 94 patients (50%), metastatic liver cancer in 76 patients (42%), perihilar cholangiocarcinoma in five patients (3%), cholangiocellular carcinoma in four patients (2%), and other disease in six patients (3%). The primary lesion of metastatic liver cancer was colon in 71 patients, stomach in two patients, pancreas in one patient, lung in one patient, and biliary tract in one patient, respectively. There was an underlying liver infection of the hepatitis C virus in 44 patients (24%) and of the hepatitis B virus in nine patients (5%). Of the 181 patients undergoing EOB-MRI of the liver in this study, 177 (97.8%) patients were classified as having the Child–Pugh grade A and four (2.2%) patients as having the Child–Pugh grade B. The median value of ICGR15 was 13.1% (range, 2.9–58.9%). The therapies provided for the liver tumors were as follows: surgical resection in 112 patients (62%), chemotherapy in 26 patients (14%), radiofrequency ablation in 11 patients (6%), transcatheter arterial chemoembolization in 13 patients (7%), proton beam radiation therapy in 12 patients (7%), and supportive care in seven patients (4%).

### Inter-evaluator variability of the LSR and correlation between the LSR and vsLSR (patient cohort 1, *n* = 24)

The intraclass correlation coefficient of the LSR amongst the four evaluators calculated using the 3D volumetric analysis system, was 0.986. The intraclass correlation coefficient between the LSR and vsLSR, evaluated by a single investigator, was 0.987.

### Correlation between the LSR and grading systems of liver function (patient cohort 2, *n* = 181)

Correlations between the LSR and grading systems of liver function were summarized in Table 1. The mean LSR was lower in the patients with the Child–Pugh grade B than those with the Child–Pugh grade A (1.57 vs 1.97, *p* = 0.014). The mean LSR was lower in the patients with the ALBI score 2 than those with the ALBI score 1 (1.64 vs 2.05, *p* < 0.001).

**Table 1:** Correlations between the liver-to-spleen ratio (LSR) and grading systems of liver function (patient cohort 2, *n* = 181)

	*n* = 181	LSR (mean)	*p*
Child–Pugh score			
A	177 (97.8%)	1.97	= 0.014
B	4 (2.2%)	1.57	
ALBI score			
1	142 (78.5%)	2.05	< 0.001
2	39 (21.5%)	1.64	

### Correlations between the LSR and laboratory data (patient cohort 2, *n* = 181)

Correlations between the LSR and laboratory data are summarized in Table 2. Positive correlations were observed between the LSR and the following parameters: platelet count (*r* = 0.307, *p* < 0.001), serum level of albumin (*r* = 0.453, *p* < 0.001), and prothrombin activity (*r* = 0.426, *p* < 0.001). Negative correlations were observed between the LSR and the following parameters: ICGR15 (*r* = − 0.601, *p* < 0.001) and serum levels of total bilirubin (*r* = − 0.370, *p* < 0.001), aspartate aminotransferase (*r* = − 0.422, *p* < 0.001), and alanine aminotransferase (*r* = − 0.287, *p* < 0.001).

**Table 2:** Correlations between the liver-to-spleen ratio (LSR) and laboratory data or histological findings

	*r*	*p*
Laboratory data (patient cohort 2, *n* = 181)		
ICGR15	− 0.601	< 0.001
White blood cell	0.067	0.368
Platelet count	0.307	< 0.001
Prothrombin activity	0.426	< 0.001
Hemoglobin	0.021	0.783
Albumin	0.453	< 0.001
Total bilirubin	− 0.370	< 0.001
Aspartate aminotransferase	− 0.422	< 0.001
Alanine aminotransferase	− 0.287	< 0.001
Creatinine	− 0.116	0.120
Histological findings (patient cohort 3, *n* = 112)		
Liver fibrosis		
METAVIR score	− 0.556	< 0.001
ROF (AZAN stain)	− 0.424	< 0.001
ROF (SMA stain)	− 0.592	< 0.001
Liver steatosis		
Kleiner grade	− 0.396	< 0.001
ROS	− 0.428	< 0.001

*ICGR15* indocyanine green retention rate at 15 min; *ROF* ratio of fibrosis; *ROS* ratio of steatosis; *AZAN* azo carmine aniline blue; *SMA* smooth muscle actin

### Correlations between the LSR and histological findings (patient cohort 3, *n* = 112)

**Fig. 4:**
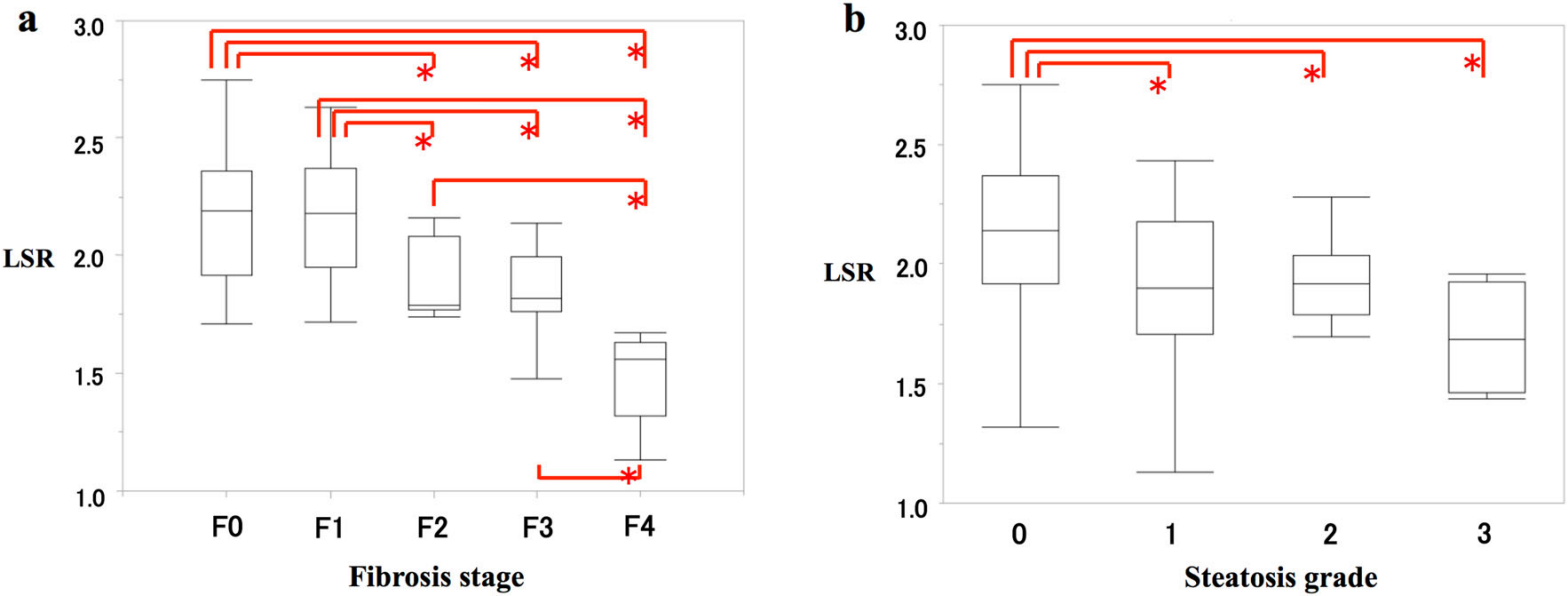
Box plots of the liver-to-spleen ratio (LSR) according to the fibrosis stage (**a**) and steatosis grade (**b**). *Significant differences in pairwise comparisons using the Mann–Whitney test

Correlations between the LSR and histological findings, including liver fibrosis and steatosis, are summarized in Table 2 and Fig. 4. In terms of liver fibrosis, negative correlations were observed between the LSR and the METAVIR score (*r* = − 0.556, *p* < 0.001), between the LSR and ROFazan (*r* = − 0.424, *p* < 0.001), and between the LSR and ROFsma (*r* = − 0.592, *p* < 0.001) (Table 2). The LSR value was significantly greater for fibrosis stages F0 and F1 than for stages F2, F3, or F4 (each pairwise comparison, *p* < 0.001) (Fig. 4a). In terms of liver steatosis, negative correlations were observed between the LSR and the Kleiner’s grade (*r* = − 0.396, *p* < 0.001) and between the LSR and ROS (*r* = − 0.428, *p* < 0.001). The LSR value was significantly greater for steatosis grade 0 than for grade 1 (*p* < 0.001), grade 2 (*p* < 0.001), or grade 3 (each pairwise comparison, *p* = 0.001) (Fig. 4b).

## Discussion

In this study, the clinical utility of the LSR, calculated as the signal intensity of the liver parenchyma divided by that of the spleen on EOB-MRI, was evaluated using a 3D volumetric analysis system. The reproducibility of the calculated LSR amongst the different evaluators was very high. The LSR was shown to be correlated with grading systems of liver function, such as the Child–Pugh score and the ALBI score. In addition, significant correlations between the LSR and ICGR15 and between the LSR and histological findings of liver fibrosis or steatosis were observed. This is the first report using a 3D volumetric analysis system of EOB-MRI to evaluate liver function.

Conventionally, signal intensity of the liver parenchyma on EOB-MRI has been measured by ROIs arbitrarily selected on a few 2D MRI slices. However, such systems might include a certain degree of selection bias of the ROIs. On the other hand, 3D volumetric analysis systems, which extract the entire volume of specific internal organs based on high-precision image processing algorithm, have been introduced in clinical settings in recent years. The benefits of preoperative simulation of liver anatomy with the use of 3D image visualization technologies have been shown in several papers [13, 14]. Takamoto et al. showed a high correlation between estimated liver volume on preoperative computed tomography and the weight of the resected specimens [15]. Ogawa et al. reported the utility of a 3D volumetric analysis system on computed tomography images to predict the area of the liver that is embolized after transcatheter arterial chemoembolization [16]. The advantage of 3D volumetric analysis systems compared to 2D ones is that they enable the calculation of the average whole liver signal intensity automatically, from only a small VOI drawn in the liver parenchyma. The present study showed that the inter-evaluator correlation coefficient of the LSR values was 0.986. This means that the LSR, calculated using a 3D volumetric analysis system of EOB-MRI, is an objective, precise, and quantitative index to measure liver signal intensity.

There are several studies that used 2D analysis systems on EOB-MRI to evaluate liver function. These studies indicated that liver signal intensity, measured from ROIs, was correlated with liver functional reserve markers, such as ICGR15, prothrombin activity, or liver fibrosis [3–5, 17]. The present study showed that the LSR calculated using a 3D volumetric analysis system on EOB-MRI was correlated with grading systems of liver function and laboratory data, such as platelet count, serum levels of albumin, total bilirubin, aspartate aminotransferase, alanine aminotransferase, prothrombin activity, and ICGR15. ICGR15 is one of the well-known biochemical indices used to predict post-hepatectomy remnant liver function. In principle, ICG is taken up to hepatocytes via the organic anion transporting polypeptide or Na^+^-taurocholate co-transporting polypeptides located in the sinusoidal membrane, and is excreted into the biliary system via the ATP-dependent export pump multidrug resistance-associated protein 2 without biotransformation [18]. Gd-EOB-DTPA is also taken up to hepatocytes via the organic anion transporting polypeptides, and is excreted into the biliary system via the multidrug resistance-associated protein 2 [19]. Therefore, in the light of the metabolic mechanism theory, the signal intensity of the liver on EOB-MRI would be expected to reflect ICGR15. The present study showed the strongest correlation between the LSR and ICGR15, compared to the other laboratory data analyzed.

There are several studies showing a significant correlation between signal intensity of the liver on 2D analysis systems on EOB-MRI and liver fibrosis [6, 20–22]. These studies evaluated liver fibrosis using either the METAVIR score or the New INUYAMA classification as categorical variables assessed by pathologists. In this study, morphometric analysis, using image processing software, enabled the quantification of fibrosis or steatosis in the liver, and showed a significantly inverse correlation between the LSR and fibrosis or steatosis. These data suggest that infiltration in the liver parenchyma of stromal tissues, such as fibrosis or steatosis, decreases the relative area of hepatocytes and leads to the reduction of signal intensity of the whole liver on EOB-MRI. Interestingly, as shown in the Supplementary Table, ICGR15 was not significantly correlated with fibrosis or steatosis in the liver. These results suggest that the function of the liver that was affected by fibrosis or steatosis was not reflected accurately by ICGR15 but by the signal intensity of the whole liver on EOB-MRI. Therefore, the LSR, calculated by a 3D volumetric analysis system on EOB-MRI, may indicate liver function more accurately than the other liver function parameters. There might be concerns regarding other components in the liver than normal parenchyma, fibrosis, and steatosis. Majority of previous studies reported no significant correlations between fibrosis or steatosis and iron deposition histopathologically or radiographically [23–25]. Moreover, there were no patients with hemosiderosis or hemochromatosis clinically in our patient cohort. Therefore, liver fibrosis or steatosis was considered to be evaluated using EOB-MRI regardless of the iron deposition.

In this study, there were five patients with perihilar cholangiocarcinoma who developed obstructive jaundice at presentation. However, we consider that the influence of obstructive jaundice on evaluating liver function using EOB-MRI was expected to be minimal, because all the patients who had obstructive jaundice were re-examined by EOB-MRI for operative indication after biliary drainage and resolution of jaundice. However, evaluation of liver function using EOB-MRI for the patients with obstructive jaundice may be a subject of future study.

Our study has several limitations. First, there was a considerable number of patients (52/304, 17%) that had to be excluded due to a different EOB-MRI acquisition protocol (mostly due to a different slice thickness). During the study period, the slice thickness of 4.6 mm might be relatively thick for 3D volumetric analysis. The MRI protocol was determined to diagnose liver tumors but not to evaluate liver function in this study. However, our study showed that the LSR was correlated with the Child–Pugh grade, the ALBI score, ICGR15 and other laboratory data, and histological findings, although the MRI conditions were set to diagnose liver tumors. These results suggest that EOB-MRI would be applied for evaluating liver function in the clinical situation. Furthermore, the MRI protocol in the previous studies that evaluated liver function using EOB-MRI did not differ widely from that in our study: the slice thickness was 5.0 mm in the study by Okada et al. [8], 4.0 mm by Nishie et al. [7], and 3.8 mm by Matsushima et al. [4]. Second, in the 3D volumetric analysis, extrahepatic-parenchymal tissues such as portal vein or hepatic vein were included in the whole liver signal intensity calculation: the LSR was the metric primarily used. However, since the study showed a high correlation between the LSR and vsLSR (LSR excluding extrahepatic-parenchymal tissues), and demonstrated that the LSR adequately reflects contrast enhancement of the liver parenchyma, this suggests that liver function can be evaluated without subtracting vessels and vascular perfusion areas. Third, two different MR scanners were used in this study. This might have caused a systemic bias in the calculation of liver or spleen signal intensities. However, the difference is not assumed to be significant, as the MRI protocols were similar between the two different machines and all images were taken by T-1 weighted technique. In a previous multicenter study, Okada et al. reported no significant differences in signal intensity of the liver on EOB-MRI between different MRI scanners [8]. Fourth, there were a large number of patients excluded for having prior history of portal vein embolization. Previous studies showed a significant decrease of signal intensity of the liver lobe after portal vein embolization [26]. Therefore, in the present study, we decided to investigate the entire liver function, instead of regional liver function. Preoperative evaluation of the regional liver function using EOB-MRI for major hepatectomy would be the subject of future study.

In conclusion, the LSR calculated using a 3D volumetric analysis system on EOB-MRI were highly reproducible, and were correlated with grading systems of liver function, laboratory data, and histological findings. EOB-MRI using a 3D volumetric analysis system may be a reliable modality to evaluate liver function.

## Electronic supplementary material

Below is the link to the electronic supplementary material.

Supplementary material 1 (DOCX 15 kb)
