# Supplementary material for: Evaluation of liver function using gadolinium-ethoxybenzyl-diethylenetriamine pentaacetic acid enhanced magnetic resonance imaging based on a three-dimensional volumetric analysis system
Source: Hepatol Int. 2018 Jun 2;12(4):368–76. doi: 10.1007/s12072-018-9874-x (PMC6096956; doi:10.1007/s12072-018-9874-x)
Supplement: Supplementary file 1 — Supplementary material 1 (DOCX 15 kb) [file 12072_2018_9874_MOESM1_ESM.docx]

**Supplementary Table** Correlations between the ICGR15 and histological findings (patient cohort 3, n=112)

|  |  | ICGR15 | |
| --- | --- | --- | --- |
|  |  | *r* | *p* |
| Liver fibrosis | METAVIR score | 0.252 | =.007 |
|  | ROF (AZAN stain) | 0.297 | =.002 |
|  | ROF (SMA stain) | 0.592 | <.001 |
| Liver steatosis | Kleiner grade | -0.074 | =.440 |
|  | ROS | -0.166 | =.081 |

ICGR15, indocyanine green retention rate at 15 min; ROF, ratio of fibrosis; ROS, ratio of steatosis; AZAN, azo carmine aniline blue; SMA, smooth muscle actin.
